# Emerging nanomedicine strategies for hepatocellular carcinoma therapy

**DOI:** 10.1002/imo2.12

**Published:** 2024-07-04

**Authors:** Chen Guo, Jiayu Zhang, Xiaomeng Cai, Rui Dou, Jiaruo Tang, Zhengyuan Huang, Xueting Wang, Yan Guo, Hanqing Chen, Jun Chen

**Affiliations:** ^1^ Department of Gastroenterology and Hepatology, Center for Medical Research on Innovation and Translation, The Second Affiliated Hospital, School of Medicine South China University of Technology Guangzhou China; ^2^ CAS Key Laboratory for Biomedical Effects of Nanomaterials and Nanosafety, Multi‐disciplinary Research Division Institute of High Energy Physics and University of Chinese Academy of Sciences (UCAS), Chinese Academy of Sciences (CAS) Beijing China; ^3^ Department of Nutrition & Food Hygiene, School of Public Health Capital Medical University Beijing China

**Keywords:** liver cancer, nanoparticles, stimulus‐responsive strategy, targeting strategy

## Abstract

Hepatocellular carcinoma (HCC) is a prevalent malignant tumor with a range of risk factors, including viral infections, alcoholic liver disease, exposure to fungal toxins, obesity, and type 2 diabetes. Despite advancements in early detection and treatment, HCC continues to exhibit a high rate of recurrence. Patients in advanced stages have a poor prognosis, and the survival rate after cancer metastasis is notably low. Consequently, there exists an urgent necessity for novel treatment strategies. Nanomedicine possesses superior attributes, such as targeted administration and responsive drug release within the tumor microenvironment. These systems demonstrate potential in HCC treatment by facilitating the transportation of diverse therapeutic drugs. This comprehensive review aims to delve into the application of nanoparticle drug delivery systems in the management of liver tumors, with particular emphasis on formulation, targeted strategies specific to liver tumors, and various methods of drug release. By offering insights into the utilization of nanoparticle drug delivery systems in the realm of liver tumor treatment, this review endeavors to assist readers in exploring their vast potential.

## Highlights

- The comprehensive review centers on the application of nanoparticle drug delivery systems in the management of liver tumors.
- It emphasizes the formulation of these drug delivery systems and targeted strategies specific to liver tumors.
- The review also explores various methods of drug release, aiming to provide valuable insights into the potential of nanoparticle drug delivery systems for liver tumor treatment.

## Graphical abstract

The review highlights the application of nanoparticle drug delivery systems in the management of liver tumors, emphasizing the formulation and targeted strategies specific to liver tumors, while also exploring various methods of drug release, aiming to provide valuable insights into the potential of nanoparticle drug delivery systems for liver tumor treatment.

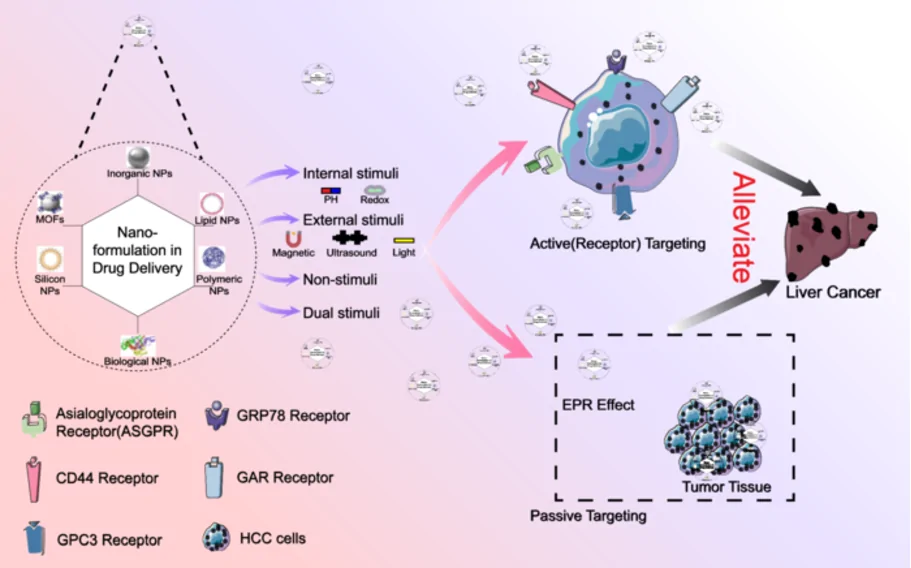

## 1 INTRODUCTION

Hepatocellular carcinoma (HCC), a malignant tumor that is increasingly prevalent, has several main risk factors including viral infections, alcoholic liver disease, fungal toxins, obesity, and type 2 diabetes [1]. Despite early‐stage diagnosis and treatment, the disease still exhibits a high recurrence rate. The prognosis becomes even more unfavorable for patients in intermediate to advanced stages, with a mere 11% 5‐year survival rate if the cancer has spread to nearby lymph nodes [2]. When the cancer metastasizes to other organs, the 5‐year survival rate drops further to only 3% [2]. There is a pressing medical requirement to explore novel therapeutic approaches for HCC due to the lack of effective treatment options available.

Nanomedicine delivery systems offer improved pharmacokinetic properties, tumor‐targeted delivery capabilities, and ‐responsive fixed‐point drug release, among other benefits. These features contribute to enhanced synergistic antitumor effects and hold significant potential for clinical applications. Nanomedicines can carry a variety of therapeutic agents, such as small molecules for chemotherapy, DNA or RNA for gene therapy, heat‐generating molecules or nanoparticles for thermotherapy, and radioisotopes for radiotherapy [3]. This versatility underscores the wide range of treatments that can be facilitated by nanomedicine delivery systems. Therefore, the utilization of nanomedicine delivery systems in HCC treatment holds great promise.

In this review, we will discuss the use of nanomedicine delivery systems in liver tumor therapy. We will start by providing a brief overview of several formulations employed in liver cancer therapy, including lipid‐based nanoparticles and nanoparticles based on natural substances, which have gained popularity in recent years. Next, we will delve into strategies for targeting liver cancer using nanomedicine, with a focus on actively targeting receptors that are overexpressed on the surface of liver tumors. Finally, we will explore different approaches for drug release, including both internal and external stimuli.

## 2 NANOMEDICINE STRATEGIES FOR HCC THERAPY

### Formulations

#### Lipid nanoparticles (LNPs)

In 2018, the US Food and Drug Administration granted approval to patisiran as the first nucleic acid drug delivered using LNP technology. Since then, LNPs have emerged as highly promising drug delivery carriers that have garnered considerable attention in the field. LNPs are composed of four lipid components, with ionizable lipids responsible for electrostatically binding to nucleic acids [4]. The functions of the other three components are as follows: cholesterol stabilizes the nanoparticle structure and enhances cellular uptake through low‐density lipoprotein receptor‐mediated endocytosis; helper lipids accelerate the structural transformation of nanoparticles within cells and promote drug release; and polyethyleneglycol (PEG) lipids enhance the overall stability of LNPs and extend their duration of circulation in the bloodstream. Due to the association of apolipoprotein E (ApoE) in serum, intravenously administered LNPs tend to accumulate predominantly in the liver, as the liver is primarily responsible for the clearance of lipoproteins bound to ApoE [5, 6].

**Figure 1:**
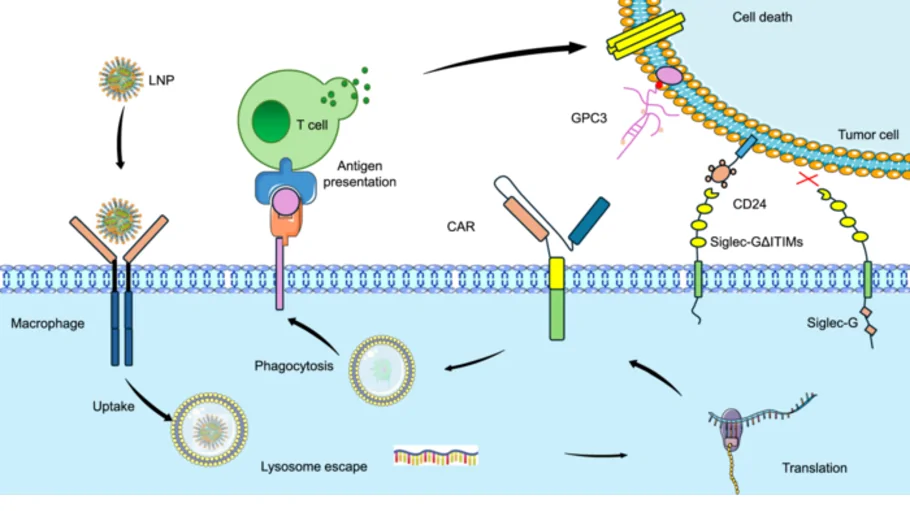
Lipid nanoparticles (LNPs) are used in the treatment of hepatocellular carcinoma (HCC). LNPs loaded with two chimeric antigen receptor (CAR) messenger RNAs (mRNAs) are utilized for the activation therapy of hepatocellular carcinoma‐associated macrophages. GPC3, Glypican‐3. Modified from reference 7, https://doi.org/10.1016/j.jconrel.2023.07.021.

The main function of LNPs is to protect the nucleic acid payload from degradation and facilitate its delivery into the cytoplasm of cells. In the field of liver cancer treatment, Yang et al. [7] have made significant progress with the development of an LNP (CAR&Siglec‐GΔITIMs LNP) (Figure 1). This LNP enables the delivery of two types of messenger RNA (mRNA) to hepatic macrophages: the first type expresses chimeric antigen receptor (CAR) specifically targeting Glypican‐3 (GPC3), enhancing macrophage phagocytosis of liver cancer cells; the second type expresses Siglec‐G CAR without ITIMs, overcoming the “don't eat me” signal. Research findings demonstrate that CAR&Siglec‐GΔITIMs LNPs exhibit excellent biocompatibility and can selectively activate macrophages to engulf liver cancer cells. This approach stimulates antigen presentation, cytokine secretion, and correction of the immunosuppressive tumor microenvironment (TME), ultimately leading to the activation of antitumor immune responses within the tumor. These LNPs significantly enhance tumor clearance rates and cytotoxicity, effectively extending the survival of mice with liver cancer [7].

Other studies rely on passive targeting to deliver encoded antigens to dendritic cells (DCs), utilizing their potent antigen‐presenting function to activate antitumor immunity. For example, Zhang et al. [4] extracted the entire RNA derived from liver cancer cells (Hepa1‐6 cells). LNPs loaded with tumor RNA were prepared using a thin‐film hydration method [4]. In both in vitro and in vivo settings, these LNPs exhibited the capacity to stimulate DC maturation, display cytotoxic effects, and exert antitumor activity. This resulted in the rapid and robust activation of antitumor immune responses, effectively preventing and inhibiting the growth of HCC.

In addition to delivering nucleic acids, LNPs are also ideal carriers for chemotherapy drugs. Doxorubicin (DOX) is an essential drug in the chemotherapy treatment of cancer; however, it exhibits cardiotoxicity, severely limiting its use in tumor treatment. Encapsulation of DOX in LNPs effectively reduces its toxicity and improves the safety of clinical administration. Duan et al. [8] aimed to enhance the therapeutic efficacy of HCC by developing a targeted treatment. They developed LNPs loaded with a pH‐sensitive DOX prodrug modified with N‐acetylgalactosamine (NAcGal) and co‐encapsulated it with sorafenib. The study found that NAcGal‐modified LNPs exhibited significantly higher cellular uptake efficiency compared to unmodified LNPs, and among all tested samples, NAcGal‐modified LNPs showed the most pronounced inhibitory effect. The results demonstrated that the LNP system exhibited remarkable synergy, optimal tumor suppression efficacy, and minimal adverse effects on the body as a whole.

#### Polymer‐based nanoparticles

Polymer‐based nanoparticles offer a wide range of synthetic advantages, allowing precise control over properties, such as size, charge, and release mechanisms. These nanoparticles have diverse functions and find applications in various fields. One significant application is their ability to be modified with ligands on their surfaces, which enables specific targeting of hepatocyte receptors and facilitates liver‐directed delivery. This liver‐targeted approach has gained widespread adoption. In a recent study, researchers successfully synthesized thiolated polymers using 1‐Ethyl‐3‐(3‐Dimethylaminopropyl)carbodiimide‐mediated conjugation reactions and lyophilization. They further developed nanoparticles using solvent diffusion and high‐pressure homogenization [9]. These nanoparticles were specifically loaded with glycyrrhetinic acid (GA), a hydrophilic bioactive compound derived from liquorice that possesses hepatocyte‐targeting properties. Additionally, GA has been shown to exhibit antihepatoma activity, making it an ideal component for this dual‐purpose system. The study demonstrated the potent cytotoxicity of the nanocomposite against liver cancer cells. Moreover, the liver‐targeting capability of the nanocomposite was confirmed through high‐performance liquid chromatography analysis of liver homogenate. This innovative approach shows great promise for advancing liver cancer treatment, providing renewed hope in the field.

**Figure 2:**
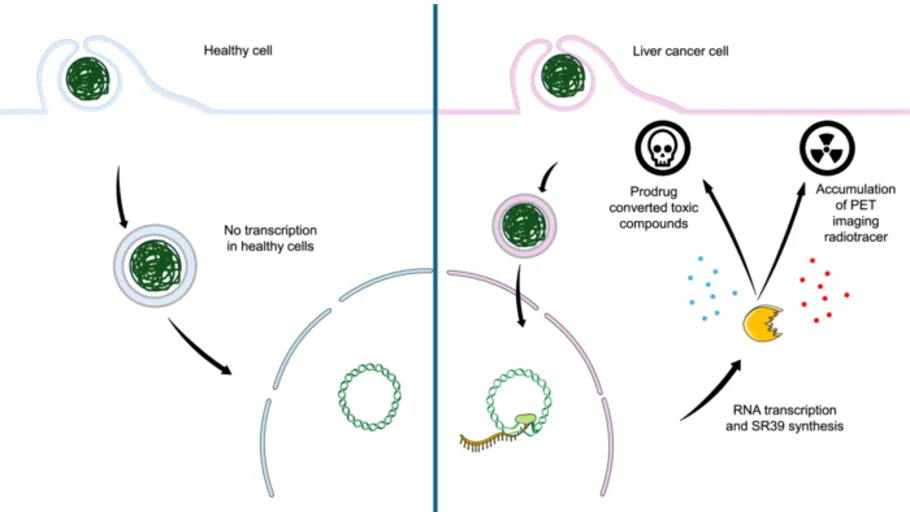
A drug delivery system capable of recognizing healthy cells and liver cancer cells. Poly (β‐Amino Esters) nanoparticles delivered a CpG‐free plasmid containing simplex virus type 1 thymidine kinase (sr39) DNA to human HCC cells, enabling selective cancer cell killing because of the expression of α‐fetoprotein in hepatocellular cells. PET, positron emission tomography. Modified from 10, https://doi.org/10.1126/sciadv.abo6406.

Additional studies have utilized differentially expressed enzymes to enable targeted therapies for HCC. For example, in a recent study, researchers utilized Poly (β‐Amino Esters) nanoparticles to transport plasmids encoding the mutated form of herpes simplex virus type 1 thymidine kinase (sr39) into liver cancer cells [10]. The sr39 thymidine kinase enables the antiviral drug ganciclovir to kill cancer cells and accumulate 9‐(4‐^18^F‐fluoro‐3‐hydroxymethylbutyl) guanine for in vivo imaging. Additionally, the plasmids delivered to liver cells were completely CpG‐free, allowing their expression to be limited to HCC cells that produce α‐fetoprotein. Through this design, specific delivery, treatment, and imaging of tumors were achieved, providing an advanced solution for targeted therapy of HCC (Figure 2).

#### Inorganic nanoparticles (INPs)

INPs are nanomaterials based on inorganic particles. Typical examples of INPs include metals, metal oxides, carbon materials, and magnetic nanoparticles. Although most of these nanoparticles are nonbiodegradable, their advantages lie in the ease of size control and surface modification, making them highly promising for applications in drug delivery systems.

Metal‐organic framework materials (MOFs), which are rapidly advancing coordination polymers with a three‐dimensional porous structure, represent an important class of porous materials. These materials possess adjustable pore sizes and high specific surface areas, making them highly suitable for drug delivery systems as they offer significant advantages, such as high drug loading capacity and ease of modification. In a previous investigation conducted by Xiao et al. [11], they employed iron (II)‐doped zeolitic imidazolate framework (ZIF‐8) nanoparticles as carriers for the encapsulation of dihydroartemisinin (DHA), an antineoplastic drug. To achieve precise delivery to cancer cells, the researchers developed a bionic system by embedding the nanoparticles into the cancer cell membrane. The surface of cancer cell membranes exhibits exceptional selectivity toward HCC tumors, facilitating precise targeting of nanoparticles. Once inside the acidic microenvironment of the cancer cells or tumor sites, DHA was triggered to release and interact with iron (II), leading to the production of reactive oxygen species (ROS) and exerting an anticancer effect. The strategy demonstrated remarkable tumor inhibition of over 90%. This MOF nanoparticle‐based approach showcases the potential of targeted drug delivery and effective cancer treatment through ROS generation.

Zhang et al. [12] introduced RF@LA‐Fe‐MOF, a novel iron (III)‐based delivery system utilizing MOF components. This system incorporated the liver‐targeting ligand and housed two small molecules, RSL3 and iFSP1. In the context of HCC, the iron (III) in RF@LA‐Fe‐MOF undergoes reduction to iron (II) triggered by glutathione (GSH). This reduction induces a ferroptosis and leads to the disassembly of RF@LA‐Fe‐MOF, thereby releasing RSL3 and iFSP1, which act as GPX4 inhibitors and FSP1 inhibitors, respectively. The concurrent inhibition of these two inhibitors substantially amplifies ferroptosis in HCC. Treatment with RF@LA‐Fe‐MOF in mice affected by subcutaneous cancer resulted in delayed tumor growth and prolonged lifespan. Moreover, in an in situ model of a C5WN1 mouse, RF@LA‐Fe‐MOF demonstrated an effective reduction of liver tumor volume and substantial inhibition of lung metastasis. This study represents a significant breakthrough as it utilizes a drug delivery system containing two small molecule ferroptosis promoters for targeted induction and enhancement of this process in HCC.

Silicon nanoparticles (SiNPs) can be prepared using the sol‐gel chemical principle by utilizing different structure‐directing agents. This enables the synthesis of SiNPs with diverse structures and morphologies, such as robust and thermally stable mesoporous silica with a high specific surface area, as well as dendritic fiber‐type silica nanoparticles with enhanced pore permeability and accessible inner particle surfaces. Additionally, it allows for the production of hollow silica nanoparticles, which offer low density, a high specific surface area, and excellent adsorption properties. Fa et al. [13] developed a core/shell material based on mesoporous silica (mSiO_2_). The external shell of this material consisted of mSiO_2_ loaded with a nitric oxide (NO) donor called S‐nitroso‐N‐acetyl‐DL‐penicillamine (SNAP) [13]. The internal core comprised upconversion nanoparticles (UCNPs) of NaYF4:Tm/Yb/Ca@NaGdF4:Nd/Yb. When the UCNPs absorbed near‐infrared (NIR) irradiation at 808 nm, they converted the NIR into ultraviolet light, triggering the release of NO from SNAP. By employing this mechanism, significant therapeutic effects were observed. The fusion of mSiO_2_ and UCNPs in this research facilitated the precise delivery of NO to liver cancer cells, leading to a marked reduction in cell viability. This novel approach shows potential for future applications in liver cancer therapy.

#### Nanoparticles based on natural substances

Extracellular vesicles or exosomes, which typically range in diameter from 30 to 200 nm are single‐membrane vesicles that are essential for intercellular communication and substance transport [14]. One of their remarkable capabilities is the delivery of bioactive components, such as nucleic acids and proteins, into the cytoplasm of recipient cells, facilitating the exchange of important substances. With their high biocompatibility, low immunogenicity, and efficient transport properties, extracellular vesicles have garnered significant attention. Cells produce extracellular vesicles under both normal and pathological conditions, underscoring their wide‐ranging sources. This broad distribution contributes to the potential clinical applications of extracellular vesicles, which can serve as valuable diagnostic biomarkers and therapeutic tools. The multifaceted nature of extracellular vesicles highlights their significance in understanding cellular communication and exploring novel avenues for medical interventions.

In recent years, there has been a significant advancement in the field of exosome‐based drug delivery platforms. These platforms have demonstrated the ability to carry and modify various therapeutic molecules, such as small molecules, nucleic acids, and protein peptides. This progress holds great promise for the development of more effective and targeted drug delivery systems. For example, the Cas9/sgRNA complex (RNP) has a large size that exceeds the loading capacity of existing delivery vehicles. However, exosomes offer a powerful solution with their remarkable loading capacity to effectively accommodate and transport the RNP. Wan et al. [15] loaded the gene‐editing protein RNP into exosomes isolated from hepatic stellate cells by electroporation to prepare ExosomeRNP, a therapeutic exosome with liver‐specific targeting and gene‐editing capabilities; in vivo experimental results demonstrated that ExosomeRNP can be selectively enriched into mouse liver and has the potential to treat multiple liver diseases including HCC.

In another study, Zou et al. [16] isolated tumor cell‐derived exosomes. The exosomes carry tumor‐associated antigens that can improve the potential of DCs to recognize HCC as a vaccine. To enhance the immunogenicity of the exosomes, the researchers attached a potent adjuvant, high mobility histone nucleosome binding protein 1, to the exosomes. This strategic modification enhanced the ability of DCs to activate T cells, thereby improving vaccine efficiency and enhancing immunotherapy for HCC. The results of this study suggest that exosomes are expected to enhance therapeutic efficacy by improving immunotherapeutic strategies.

Serum albumin, the predominant protein in the blood, plays a vital role in maintaining colloid osmotic pressure and regulating plasma pH. Serum albumin offers several advantages for drug delivery in cancer therapeutics [17]. First, it exhibits high biocompatibility, biodegradability, non‐immunogenicity, and clinical safety. Second, the chemical structure and conformation of albumin allow it to interact with a wide range of drugs, potentially protecting them from elimination and metabolism in the body, thereby improving their pharmacokinetic properties. Lastly, albumin can interact with receptors overexpressed in many diseased tissues and cells, providing unique functionality for actively targeting disease sites without the need for specific ligands to be added to nanocarriers. Drug molecules such as Warfarin, Ibuprofen, Paclitaxel (PTX), and others can be incorporated into the cavities of different structural domains of serum albumin or noncovalently bound to the protein through hydrophobic and electrostatic interactions. This enables their delivery in vivo utilizing serum albumin as a carrier. Most antitumor drugs suffer from drawbacks, including poor water solubility, high toxicity, and rapid breakdown and metabolism within the body. By loading these drugs into albumin‐based nanoparticles, the issues of poor water solubility and sensitization can be effectively addressed. Thao et al. [18] conducted a study where they synthesized Lac‐BSA by coupling lactose with bovine serum albumin (BSA). They successfully prepared well‐dispersed DOX/Pac Lac‐BSA nanoparticles, loaded with DOX and PTX, using high‐pressure homogenization. These nanoparticles not only demonstrated synergistic cytotoxic effects of DOX and PTX in HepG2 cells but also exhibited enhanced liver localization in mice. Based on these findings, the researchers suggested that lactose‐modified albumin‐based nanoparticles have the potential to serve as therapeutic vehicles for HCC treatment, utilizing hepatocyte targeting to achieve effective therapeutic outcomes.

In a study by Wu et al. [19], glycyrrhizic acid was combined with human serum albumin and used to encapsulate resveratrol within nanoparticles via high‐pressure homogenization emulsification. These nanoparticles, referred to as glycyrrhizic acid‐coupled human serum albumin nanoparticles, had an average size of 108.1 ± 5.3 nm with a very uniform particle size distribution. The researchers observed that these nanoparticles showed a higher uptake rate of resveratrol compared to pure resveratrol and the uptake rate increased with higher nanoparticle concentrations. Additionally, these resveratrol‐carrying nanoparticles demonstrated effective liver tumor targeting and sustained release properties.

Ferritin, a widely distributed protein in living organisms, plays a crucial role in maintaining iron homeostasis in the body and acts as a cellular antioxidant. It is an endogenous, naturally occurring protein in the human body and is typically composed of 24 separate subunits in eukaryotic cells. Its unique protein cage structure and self‐assembly properties provide innovative concepts for drug carriers [20]. Jiang et al. [21] developed the drug nanocarrier HccFn‐DOX. Initially, they demonstrated the presence of the glucose regulatory protein 78 (GRP78)‐targeting peptide SP94 on the outer surface of anserine ferritin Fn (HccFn) using genetic engineering methods, followed by loading DOX into the cavity of HccFn [21]. The experimental results revealed that ultrahigh doses of DOX were encapsulated in the HccFn nanocapsules. In comparison to free DOX, HccFn‐DOX significantly reduced the impact of chemotherapy drugs on healthy organs, resulting in a sixfold increase in the maximum tolerated dose.

Nucleic acids offer an ideal platform for a variety of unique applications, leveraging their synthetic maneuverability, high sensitivity and affinity, and straightforward framework modification. DNA origami, a cutting‐edge technology for creating intricately complex nanopatterns or structures using DNA strands and short DNA fragments, was initially proposed by Rothumend in 2006 [22]. DNA origami technology enables the manipulation and organization of molecules at precise nanoscale locations with active targeting, excellent biocompatibility, and biostability. Jiang et al. [23] developed adriamycin‐loaded DNA origami structures in two‐dimensional and three‐dimensional forms, and applied DNA nanostructure–adriamycin complexes to a subline of DOX‐resistant cells (res‐MCF 7). The experimental results demonstrated that free adriamycin and loaded adriamycin double‐stranded DNA were ineffective in killing res‐MCF 7. In contrast, the same concentration of origami‐bound adriamycin induced cell death. This led to the hypothesis that DNA nanostructured vectors have the potential to overcome adriamycin resistance.

### Liver‐specific targeting strategy

Traditional antitumor drugs suffer from drawbacks, such as inadequate biodistribution, pharmacokinetics, poor target selectivity, high drug resistance, and significant toxicity to nontargeted tissues. HCC typically presents a prolonged latency period, resulting in most patients being diagnosed at intermediate or advanced stages, necessitating chemotherapy as the primary treatment [24]. However, chemotherapy often leads to severe side effects, possibly due to the non‐selectivity of highly toxic conventional chemotherapeutic agents for cancer cells, causing harm to normal cells and limiting the accumulation of active chemotherapeutic agents at the HCC site, resulting in insufficient drug concentrations to effectively combat HCC. Furthermore, multidrug resistance frequently arises during HCC chemotherapy, leading to cancer recurrence and diminished quality of life. In comparison to conventional chemotherapeutic agents, targeted therapies exhibit greater specificity for tumor tissues and enhanced efficacy in eradicating cancer cells. Moreover, upon introduction into the body, nanoparticles typically accumulate in the liver based on their properties [25].

Nanocarriers identify tumor regions by physicochemical differences between tumor and normal cells. Passive targeting is also referred to as the “enhanced permeability and retention (EPR) effect.” The EPR effect indicates that molecules or particles of specific sizes have a tendency to accumulate in tumor tissue more than in normal tissue, thereby indirectly pinpointing the tumor's location. Active targeting, which determines tumor location through surface receptors overexpressed by tumor cells, mainly refers to conferring the ability of a drug or its carrier to actively bind to a target. In practice, both targeting strategies are often executed concurrently, with ligand‐modified nanodrugs initially depending on passive uptake mechanisms in the liver before ligand‐mediated cellular internalization takes place [26].

#### Passive targeting

Passive targeting utilizes the inherent properties of drug carriers to achieve targeting through natural phagocytosis in the body. It capitalizes on the EPR effect: the endothelial gap of normal microvessels is dense and structurally intact, impeding the passage of macromolecules and particles through the vascular wall. In contrast, solid tumor tissues feature more newly formed blood vessels, wider gaps in the vascular wall, a relatively unstable structure, and a lack of lymphatic drainage. Passive targeting leverages this disparity to facilitate the selective distribution of macromolecular drugs in tumor tissues, thereby enhancing drug efficacy and reducing systemic side effects.

#### Active targeting

The main objective of active targeting is to confer the active binding ability of a drug or its carrier to the target. Specific targeting of receptors overexpressed on the surface of tumors through surface functionalization of nanomaterials and ligand‐mediated approaches directs modified nanoparticles to target organs, tissues, and cells, achieving low toxicity and high therapeutic efficacy during drug delivery [27, 28, 29].

Asialoglycoprotein receptor (ASGPR) is an endocytic receptor expressed on the surface of hepatocytes and previous studies have confirmed its presence in HCC [30]. It is abundantly expressed on hepatocytes and has limited expression elsewhere in the body. ASGPR can facilitate the internalization of macromolecules through lattice‐protein‐mediated endocytosis. Asialofetuin, an asialoglycoprotein containing terminal galactose residues, is widely utilized for liver targeting purposes. Epirubicin (EPI), an anthracycline derivative, is a key chemotherapy drug used in the treatment of HCC. However, its long‐term administration is hindered by severe side effects, such as cardiomyopathy and congestive heart failure. To address this challenge, Nasr et al. [31] developed a delivery strategy aimed at enhancing the effectiveness of chemotherapy drugs while reducing cardiotoxicity. They achieved this by loading EPI into chitosan‐poly(lactic‐co‐glycolic acid nanoparticles that were conjugated with asialofetuin, forming EPI nanoparticles. This nanoparticle formulation enabled the selective targeting of hepatocytes. Compared to free EPI, EPI nanoparticles demonstrated significantly improved antiproliferative effects, as observed in the HepG2 cell line. EPI nanoparticles showed positive results in the assessment of cardiac toxicity. They reduced the levels of the pro‐inflammatory cytokine tumor necrosis factor‐α, indicating a reduction in oxidative stress‐induced cardiac toxicity. Additionally, the decrease in levels of lipid peroxidation products and NO further confirmed this. Ultimately, this therapy based on biodegradable nanoparticles, which specifically target hepatocytes, not only demonstrated significant efficacy but also exhibited higher safety compared to traditional methods.

CD44, a receptor that binds to Hyaluronic acid (HA) and facilitates cellular adhesion to extracellular matrix ligands, exhibits upregulated expression in fibrosis while being comparatively downregulated in the healthy liver. Targeting drugs to hepatocytes that overexpress CD44 holds the potential for reversing liver fibrosis and inhibiting the development of HCC. HA, which serves as the main ligand for CD44, has been utilized in the advancement of nanocarriers that exhibit tumor‐targeting properties and improve cellular uptake [32].

**Figure 3:**
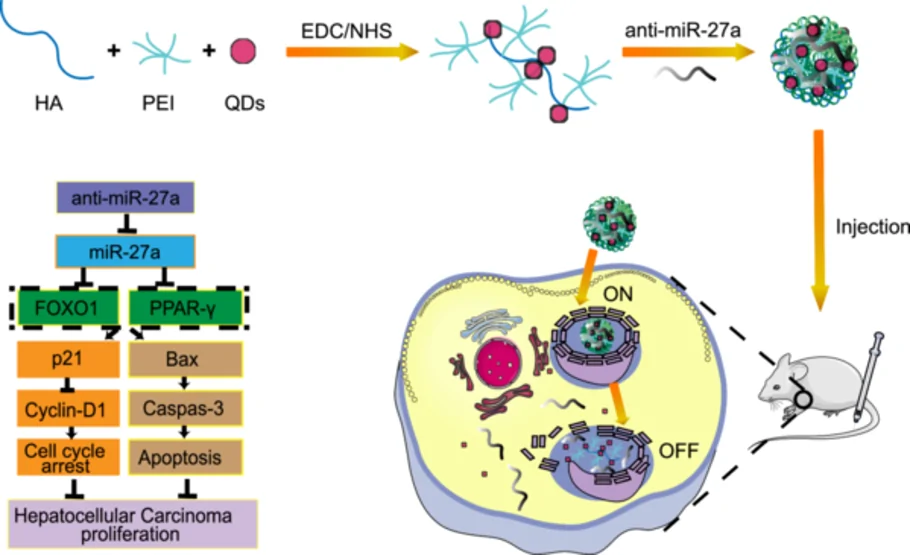
Nanodelivery system for hepatocellular carcinoma therapy targeting CD44 via hyaluronic acid (HA). A multifunctional nanoparticle utilizing HA for CD44 targeting was developed for noninvasive tracking of targeted delivery of anti‐miR‐27a oligonucleotides against liver cancer. The nanoparticle exhibited effective and selective anticancer effects in vitro and in vivo, downregulating oncogenic transcription factors FOXO1 and peroxisome proliferator‐activated receptor while enabling tumor imaging and tracking of micro RNA‐based modulation therapy. EDC/NHS, 1‐(3‐dimethylaminopropyl)‐3‐ethyl carbodiimide/N‐hydroxysuccinimide; PEI, polyethylenimine; QD, quantum dots. Modified from reference 34, https://doi.org/10.7150/thno.25255.

To enhance the hepatoprotective activity of Forsythiaside A (FA), Gong et al. [33] developed HA‐mExo‐FA, a nanocarrier system that encapsulates FA within exosomes. The exosomes were modified with HA to facilitate targeted delivery to cells overexpressing CD44. This approach effectively improved the utilization efficiency of FA. In vitro studies demonstrated that HA‐mExo‐FA reduced the expression levels of α‐smooth muscle actin and collagen genes, inhibited transforming growth factor‐β1‐induced LX2 cell proliferation, and induced apoptosis in activated LX2 cells [33]. Zheng et al. [34] explored the application of antisense oligonucleotides in liver cancer treatment. They developed a nanocarrier platform using HA modified with polyethyleneimine (PEI) and NIR fluorescent quantum dots (QDs) (Figure 3). The platform efficiently encapsulated anti‐miR‐27a oligonucleotides through electrostatic interactions, forming anti‐miR‐27a/QD‐HA‐PEI complexes. In both in vitro and in vivo studies, this platform demonstrated selective anticancer effects by directly downregulating oncogenic transcription factors FOXO1 and peroxisome proliferator‐activated receptor. Moreover, the platform enabled fluorescence quenching in the cellular environment, indicating the successful release of the antisense oligonucleotides. These findings demonstrate the potential of dual‐fluorescent nanoparticles as a versatile platform for miRNA‐based therapy, enabling simultaneous tumor imaging and therapeutic monitoring. This approach holds promise for the development of effective and safe strategies in the treatment of liver cancer [34].

GPC3 is a protein with a total length of 65 kDa and 580 amino acids. It is an antigenic protein that is specifically associated with HCC, being expressed in tumor cells of embryonic origin and HCC patients [35]. GPC3 is rarely observed in normal adult tissues, as well as in pathological liver conditions, such as steatotic liver, cirrhosis, hepatitis, or liver injury. Therefore, GPC3 has been widely recognized as an excellent diagnostic and therapeutic target for HCC [36]. Notably, Feng et al. [37] employed phage display technology to identify a specific peptide, ALLANHEELFQT, which targets GPC3. This peptide was employed to modify the surface of nanocarriers, exhibiting exceptional tumor tissue targeting ability and preserving the integrity of normal tissues [38]. Through the SELEX process, Park et al. [39] performed a screening of aptamers targeting GPC3 and incorporated the chemotherapeutic agent gemcitabine into an aptamer called G12msi. This internalization of gemcitabine into the aptamer demonstrated their successful approach [39]. They assessed the antitumor activity of G12msi using in vitro and in vivo models and observed that G12msi exhibited high specificity in binding to GPC3‐overexpressing HCC tumor cells, along with effective internalization. Furthermore, treatment with G12msi resulted in the inhibition of cell proliferation in HCC cell lines expressing GPC3, while demonstrating minimal cytotoxicity toward control A431 cells. The administration of G12msi via systemic delivery notably suppressed tumor growth in HepG2 cells without causing any observed toxicity, underscoring the potential of utilizing gemcitabine based on the GPC3 aptamer as a promising therapeutic approach for treating HCC.

GRP78 belongs to the heat shock protein 70 family. It functions as an endoplasmic reticulum chaperone protein and plays a crucial role in protein folding and trafficking within the ER. Moreover, GRP78 serves as a marker for ER stress. In tumor cells, GRP78 is often upregulated and accumulates on the cell membrane, facilitating immune evasion and conferring resistance to apoptosis and chemotherapy. The overexpression of GRP78 on the cell surface is associated with aggressive and metastatic tumor phenotypes, leading to poor prognosis and high recurrence rates. Jiang et al. [21] identified that the SP94 peptide specifically binds to GRP78 through chromosomal immunoprecipitation technique. Based on this finding, they designed a targeted drug delivery system called HccFn‐DOX nano‐cages. These nano‐cages consist of ferritin, where the outer surface is modified with the SP94 peptide, and the cavity is loaded with the drug DOX. The HccFn‐DOX nano‐cages actively target GRP78 on the cell surface and passively target tumor cells through the EPR effect. With its high loading efficiency of DOX, this nanocarrier proves to be highly effective for drug delivery against HCC.

GA receptor (GAR) is the receptor of GA. GA is derived from the hydrolysis of glycyrrhizin (GL) and is extracted from licorice, a traditional Chinese medicine used to treat hepatic diseases [40]. Both GA and GL have been utilized for their therapeutic effects on the liver. Research has shown that hepatocytes possess highly specific binding sites for GA and GL on their cellular membranes, with GA exhibiting a significantly higher number of binding sites compared to GL. It has been reported that GAR is abundantly expressed on the sinusoidal surface of hepatocytes, with expression levels 1.5–4 times higher than those in normal tissue. Therefore, targeting hepatocytes by GA is a very promising therapeutic approach. The following two examples are used to illustrate how GA can be used.

Resveratrol is a natural compound with anti‐inflammatory, antimicrobial, and potential anticancer activities. Nevertheless, its solubility is limited, restricting its utilization. Studies have shown that human serum albumin nanoparticles conjugated with GA can enhance the solubility of resveratrol and provide sustained and slow drug release [19]. Fluorescence spectra of FITC‐labeled samples indicate that HepG2 cells exhibit more effective absorption of human serum albumin‐GA nanoparticles loaded with resveratrol compared to nanoparticles without GA.

Curcumin (Cur), a phenolic antioxidant derived from the turmeric plant, possesses various therapeutic properties. However, the low water solubility and poor bioavailability of this compound impede its clinical application. To address this limitation, Chen et al. [41] developed a novel formulation called GA‐modified Cur supramolecular pre‐gelator. The GA modification led to increased cellular uptake and enhanced growth inhibition of liver tumor cells, leveraging the overexpression of GA receptors in HCC. These improvements were observed in comparison to the control group. This study introduces a promising nanomaterial for liver tumor chemotherapy.

### Stimulus‐responsive strategies

**Figure 4:**
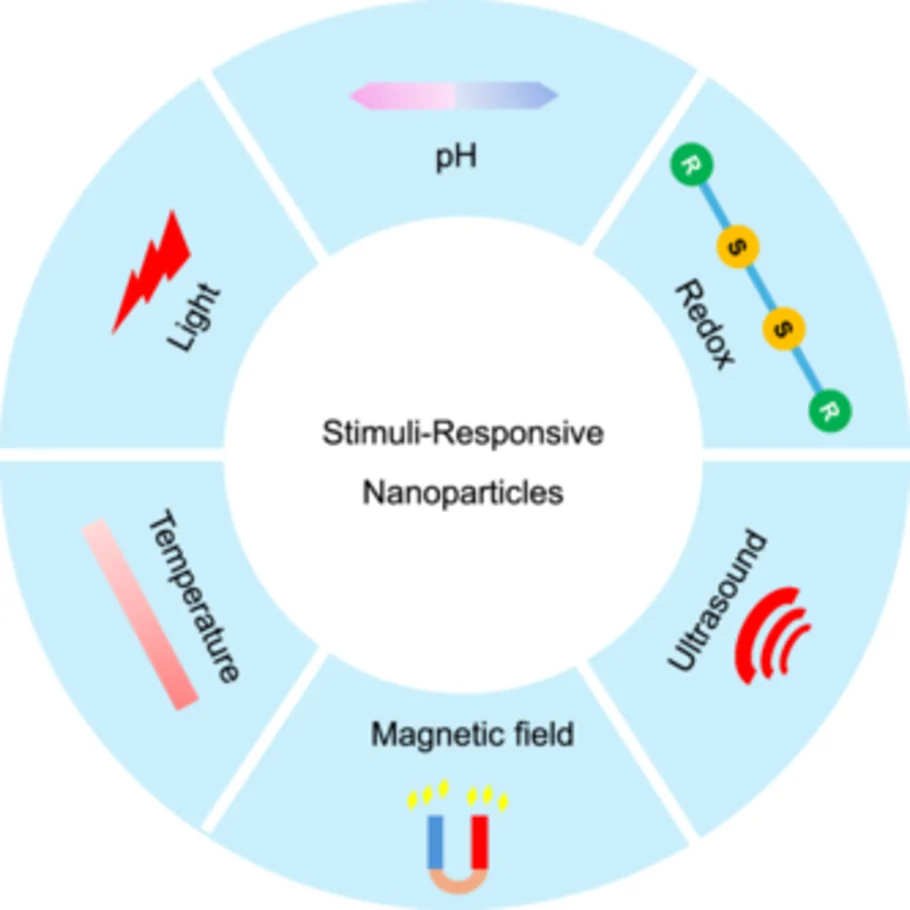
Factors for stimuli‐responsive release strategies. Factors influencing stimuli‐responsive release strategies include light, pH, redox potential, temperature, magnetic field, and ultrasound. These stimuli can be harnessed to trigger the controlled release of therapeutic agents from drug delivery systems, offering potential applications in targeted and responsive drug delivery. Modified from reference 27, https://doi.org/10.1186/s12951-022-01309-9.

Stimulus‐responsive nanocarriers can dynamically adjust their physical behavior or chemical structure according to subtle changes in the external environment. This capability enables precise and controlled drug release at the tumor site, ensuring a fixed location, quantity, and timing of drug delivery. By doing so, it minimizes potential harm to normal tissues while maximizing the therapeutic effectiveness of the drug. This breakthrough has led to significant advancements in tumor research and diagnosis. Stimulus‐responsive nanocarriers can be targeted through a variety of stimuli, including external factors, such as light, ultrasound, or magnetic fields, as well as internal cues like pH and tissue microenvironment (Figure 4) [27]. These stimuli facilitate the release of drugs from nanoparticles specifically on the targeted cells, resulting in highly efficient therapeutic outcomes.

#### Responsive nanomaterials for internal stimuli

Tumor tissues exhibit a different cellular microenvironment due to their unique physiological characteristics. Due to the notable acidity of the TME in comparison to normal tissues, pH‐responsive nanoparticles have gained considerable attention in cancer immunotherapy. The high acidity of the TME, as opposed to normal tissues, has sparked extensive interest in pH‐responsive nanoparticles for cancer immunotherapy. Biopharmaceutical formulations with pH‐sensitive carriers are able to respond to subtle pH changes, promote endosomal escape, and avoid degradation of nucleic acids or proteins in lysosomes [42].

GSH is pivotal in microenvironment regulation. The concentration of GSH in tumor tissues has been found to be at least four times higher than that in normal tissues and it is particularly elevated in certain multidrug‐resistant tumors. This disparity in the reducing environment between tumor cells and healthy cells opens up the possibility of designing nanodrug carriers [43, 44]. Hence, we have designed amphiphilic copolymers that can be reducibly degraded, utilizing poly (β‐amino ester)‐g‐poly(ethylene glycol) as the core component. In an aqueous solution, these copolymers undergo self‐assembly to generate micellar nanoparticles with a core‐shell structure that effectively encapsulates DOX. Confocal microscopy analysis of the human HepG2 cell line reveals rapid internalization of DOX and effective release in the cytoplasm. Compared to nonreducible micelles, this reducible micelle exhibits lower cytotoxicity [45].

We conducted an additional study with the aim of enhancing the effectiveness of cancer drugs by developing pH and reduction‐responsive bis‐corresponding nanoclusters. These innovative copolymers were synthesized via Michael addition polymerization, employing 2,2′‐dithiodiethanol diacrylate, 4,4′‐trimethylene dipiperidine, and methoxy‐PEG‐NH2. The micelles exhibited enhanced DOX release in response to a mildly acidic environment (pH 6.5), leading to accelerated drug release compared to higher pH levels or the presence of a reducing agent DTT at concentrations of 5 mM.

Furthermore, we observed that these micelles exhibited rapid internalization, facilitating efficient drug delivery to the nucleus and effectively inhibiting cell growth. This approach has shown promising results in HepG2 tumor cells, highlighting the potential of these pH and reduction dual‐sensitive nanocarriers as a novel and effective strategy [46].

#### Responsive nanomaterials for external stimuli

Magnetic fields, for instance, have a lower impact on biological tissues, making them ideal for selectively controlling drug accumulation and release in target tissues when combined with magnetic nanoparticles. This approach significantly reduces the impact on surrounding tissues [47]. Superparamagnetic iron oxide nanoparticles (SPIONs) possess high magnetic stability and low toxicity, making them suitable for relevant therapy when combined with magnetic fields. This combination enhances the therapeutic effectiveness by increasing the localized drug concentration at the target site, overcoming challenges associated with conventional therapies. A study by You et al. [48] developed a delivery system called PEG‐PCL‐PEI‐C14‐SPIONs (PPPCSs), which effectively protected circ_0058051 small interfering RNA (siRNA) from degradation in serum and efficiently delivered siRNA to human liver cancer cells (SMMC‐7721). Intravenous injection of PPPCSs/siRNA complexes successfully suppressed tumor growth in a subcutaneous tumor model [48].

Light‐based therapies have emerged as a promising approach in tumor therapy. Phototherapy commonly employs photosensitizers that exhibit low toxicity in the absence of light but selectively induce cancer cell death upon light exposure, minimizing damage to normal tissue. In addition to the precise targeting of tumors with carefully designed photosensitizers, spatial control of light can be implemented to irradiate the tumor site while minimizing harm to healthy tissue. This dual selectivity offered by phototherapy significantly reduces systemic toxicity associated with conventional chemotherapy or radiation therapy approaches [49].

Phototherapy primarily utilizes NIR with deep penetration capabilities and can be classified into three main types. First, photothermal therapy (PTT), in which exposure to light generates heat, directly leading to the death of cancer cells. Second, photodynamic therapy activates a photosensitizer to produce ROS. This generated ROS is responsible for killing the cancer cells. Lastly, light‐induced heat can trigger the release of encapsulated drugs, resulting in the destruction of cancer cells. These three therapies are often used in combination. Du et al. [50] have developed a novel nanoparticle drug delivery system known as NO‐DOX@PDA‐TPGS‐Gal. This system features a core/shell structure composed of d‐α‐tocopheryl PEG 1000 succinate (TPGS)‐galactose (Gal)/polydopamine (PDA). By leveraging the photothermal properties of dopamine, localized temperature elevation can be achieved through NIR irradiation. This not only facilitates the release of DOX but also triggers the heat‐induced release of NO from N,N′‐di‐sec‐butyl‐N,N′‐dinitroso‐1,4‐phenylenediamine (BNN). Consequently, this innovative approach enables a dual therapy strategy combining chemotherapy and PTT for the treatment of HCC. The chemo‐PTT approach utilizing NO‐DOX@PDA‐TPGS‐Gal has demonstrated remarkable efficacy against drug‐resistant HCC cells both in vitro and in vivo. Importantly, it has exhibited significant potential in prolonging the lifespan of mice with drug‐resistant tumors, indicating its promising therapeutic value.

Ultrasound is a periodic oscillating mechanical wave with a frequency exceeding the range of human hearing (>20 kHz). In medicine, ultrasound waves with frequencies between 20 and 100 kHz are defined as low‐frequency ultrasound, which is commonly used to initiate sonoporation and mechanically disrupt drug carrier leakage. Ultrasound‐targeted microbubble disruption is a technology that combines ultrasound with a functional carrier, providing distinct advantages in the delivery of chemotherapy for solid tumors. Guo et al. [51] designed ultrasound‐sensitive nanobubble (NB) drug carriers called FTY720@SPION/PFP/RGD‐NBs. The carrier has an RGD‐modified liposome as the shell, with Perfluoropyridine (PFP), SPION, and fingolimod (FTY720) encapsulated inside. Under ultrasound mediation, it exhibited an enhanced drug release rate. Cellular uptake experiments confirmed that this drug carrier effectively accumulated in HCC, particularly with the assistance of low‐intensity focused ultrasound [51].

#### Responsive nanomaterials for dual internal and external stimuli

**Figure 5:**
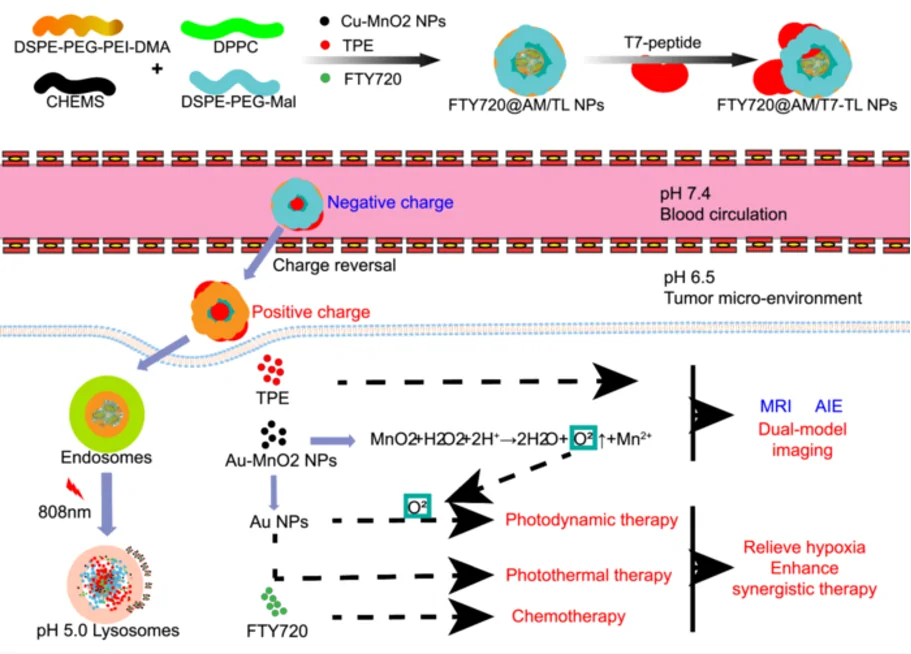
Dual stimulation with near‐infrared (NIR) and acidity for liver cancer treatment and imaging. An all‐in‐one therapeutic nanoplatform (FTY720@AM/T7‐TL) is designed for hepatocellular carcinoma treatment, featuring gold‐manganese dioxide nanoparticles, tetraphenylethylene, fingolimod (FTY720), hybrid liposome, and T7 peptides. The platform's pH‐responsive charge‐reversal property enhances blood stability and cellular uptake, enabling sustained drug release and in situ O_2_ generation for improved photodynamic therapy. In vitro and in vivo studies demonstrate its potential for dual‐modal imaging‐guided synergistic therapy of HCC. AIE, aggregation‐induced emission imaging; CHEMS, cholesteryl hemisuccinate; DMA, 2,3‐dimethylmaleic anhydride; DPPC, 1,2‐dipalmitoyl‐sn‐glycero‐3‐phosphocholine; DSPE, 1,2‐distearoyl‐sn‐glycero‐3‐phospho‐ethanolamine; Mal, maleimide; MRI, magnetic resonance imaging; NPs, nanoparticles; PEG, polyethyleneglycol; PEI, polyethylenimine; TPE, tetraphenylethene. Modified from reference 52, https://doi.org/10.1016/j.mtbio.2022.100338.

In the design of nanocarriers, a combination of external and internal stimuli is often utilized to achieve optimal drug release. For instance, Chen et al. [52] developed a conveniently integrated nanoplatform named FTY720@AM/T7‐TL for HCC (Figure 5). This platform achieves charge reversal in acidic environments and drug release under NIR irradiation. Specifically, the nanoplatform consists of gold‐manganese dioxide (Au‐MnO_2_) nanoparticles, which serve as multifunctional components, including photosensitizers, photothermal agents, peroxidase enzyme catalysts, and T1 magnetic resonance imaging agents; tetraphenylethene (TPE), used as a fluorescent imaging agent; and FTY720, used as a chemotherapeutic drug. These components are encapsulated in hybrid liposomes. Upon entry into the TME from the bloodstream, the acidic microenvironment causes the nanoplatform's charge to switch from negative to positive, facilitating its engulfment. Once inside the cells, the nanoplatform releases the internally carried Au‐MnO_2_, TPE, and FTY720 upon NIR stimulation. This dual‐stimulus effect, both internal and external, is advantageous in two ways: first, it ensures that the drugs primarily bind with cancer cells in the slightly acidic TME, and second, it minimizes damage to normal tissues by controlling the range of NIR irradiation.

Du et al. [50] developed a new nanosystem, NO‐DOX@PDA‐TPGS‐Gal, which involved TPGS‐Gal loaded with DOX and NO donor BNN coated with PDA. This nanosystem exhibits the ability to selectively target hepatocytes through Gal‐ASGPR‐mediated recognition and release DOX in the vicinity of HCC cells within the lower acidic pH microenvironment of tumors. Additionally, the use of NIR‐assisted photothermal conversion accelerated the release of DOX and initiated NO production within the BNN. The combined chemo‐PTT with NO‐DOX@PDA‐TPGS‐Gal demonstrated synergistic effects of DOX, NO, and heat, displaying potent anticancer activity both in vitro and in vivo against drug‐resistant HCC cells and significantly extending the lifespan of drug‐resistant hormonal mice.

## 3 CONCLUSION AND FUTURE PERSPECTIVES

HCC is a malignant tumor that seriously threatens human health and has always been a focus of attention in the medical field. Treatment of HCC has brought new hope with the development of nanotechnology. Nanomedicine, as a new drug delivery system, can encapsulate chemotherapy drugs into nanoparticles and achieve targeted delivery and controlled release, thereby increasing drug accumulation in tumor cells and prolonging circulation time in the body.

Some nanomedicines have shown significant efficacy in clinical trials, such as Doxorubicin‐Transdrug™ and Thermodox®. These examples demonstrate the broad prospects of nanomedicine in the treatment of HCC. However, there are still challenges in the clinical translation of nanomedicine. Minor changes can alter its biocompatibility and toxicity, requiring more research to ensure its safety and efficacy. In addition, it is necessary to enhance the understanding and application of nanomedicine by clinical practitioners to better utilize it in clinical practice. To further improve the application of nanomedicine in the treatment of HCC, research on the characteristics of nanomaterials should be strengthened to ensure their stability and safety. Additionally, research on the combined application of nanomedicine with other treatment methods is needed to explore more treatment possibilities. In summary, nanomedicine as an emerging treatment method has shown tremendous potential in the treatment of HCC, but further research and clinical practice are needed to address the challenges it faces to better benefit patients.

## AUTHOR CONTRIBUTIONS

**Chen Guo**: Writing—original draft; conceptualization; investigation; visualization; validation. **Jiayu Zhang**: Writing—original draft; writing—review and editing; visualization; validation; investigation; conceptualization. **Xiaomeng Cai**: Visualization. **Rui Dou**: Visualization. **Jiaruo Tang**: Investigation; validation. **Zhengyuan Huang**: Investigation; validation. **Xueting Wang**: Investigation; validation. **Yan Guo**: Investigation. **Hanqing Chen**: Funding acquisition; project administration; supervision. **Jun Chen**: Conceptualization; supervision; funding acquisition; writing—review and editing.

## CONFLICT OF INTEREST STATEMENT

The authors declare no conflict of interest.

## ETHICS STATEMENT

No animals or humans were involved in this study.

## Data Availability

No new data and scripts were generated in this review. Supporting Information (graphical abstract, slides, videos, Chinese translated version, and updated materials) is available online, DOI or http://www.imeta.science/imetaomics/.
